# Supplementary material for: COUnty aggRegation mixup AuGmEntation (COURAGE) COVID-19 prediction
Source: Sci Rep. 2021 Jul 12;11:14262. doi: 10.1038/s41598-021-93545-6 (PMC8275764; doi:10.1038/s41598-021-93545-6)
Supplement: Supplementary file 1 — Supplementary Information. [file 41598_2021_93545_MOESM1_ESM.pdf]

Supplementary Information for
COUnty aggRegation mixup AuGmEntation (COURAGE) COVID-19 Prediction

Siawpeng Er, Shihao Yang, Tuo Zhao
Correspondence to: shihao.yang@isye.gatech.edu, tourzhao@gatech.edu

This PDF file includes:

- Supplementary Table S1
- Supplementary Figure S1

Supplementary Table

Model	Week 1	Week 2	Average
Mixup	70.4711	85.9666	78.2188
**COURAGE**	70.8875	87.6565	79.2720
Ensemble[18]	67.2675	93.3830	80.3252
Karlen[33]	69.1216	94.2766	81.6991
UMass-MB[43]	66.5562	100.0608	83.3085
MOBS[45]	68.9179	99.7629	84.3404
Oliver Wyman[40]	69.0729	103.6900	86.3815
County	74.3374	98.5684	86.4529
GT-DeepCOVID[15]	76.1277	102.5137	89.3207
Naive	81.9271	107.9848	94.9559
USC[46]	82.1277	114.5714	98.3495

Table S1. Comparison among different models for average MAE (from 2020-12-19 to 2021-02-06). When new trend data is the main contribution of total prediction period, the mixup data augmentation helps to improve the model's accuracy.

Supplementary Figure

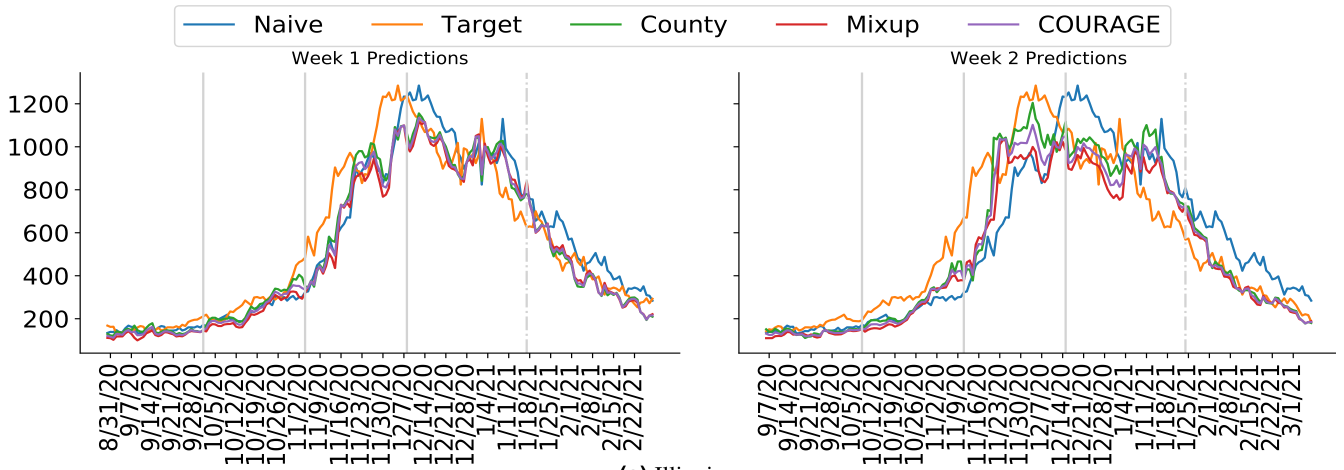

(a) Illinois

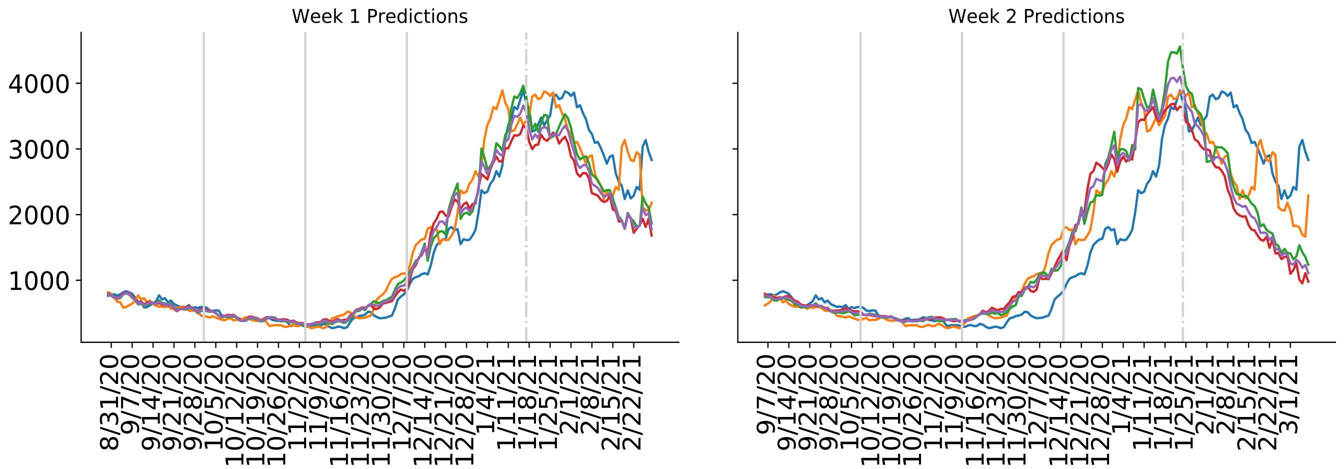

(b) California

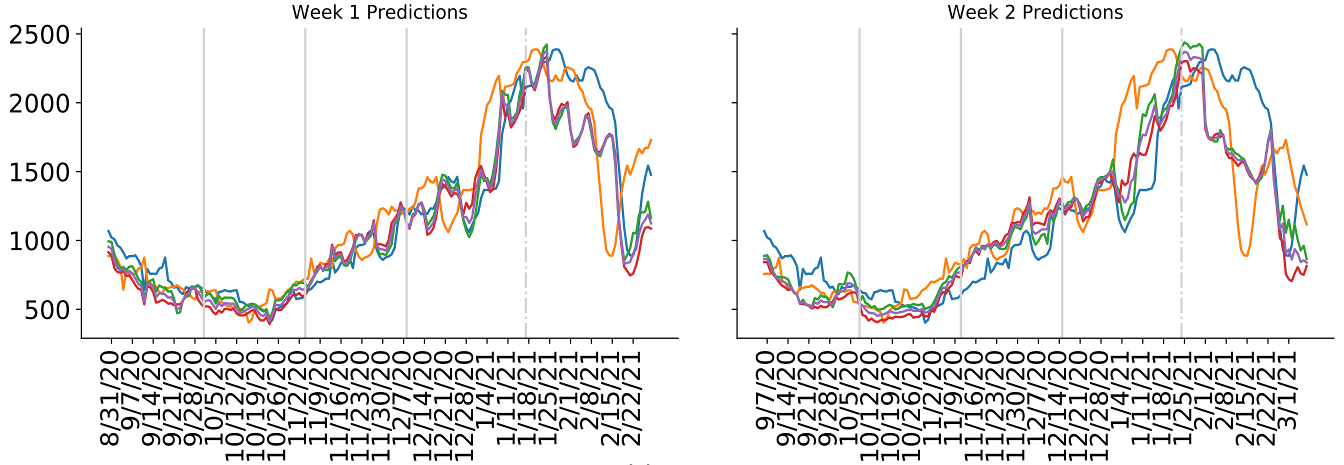

(c) Texas

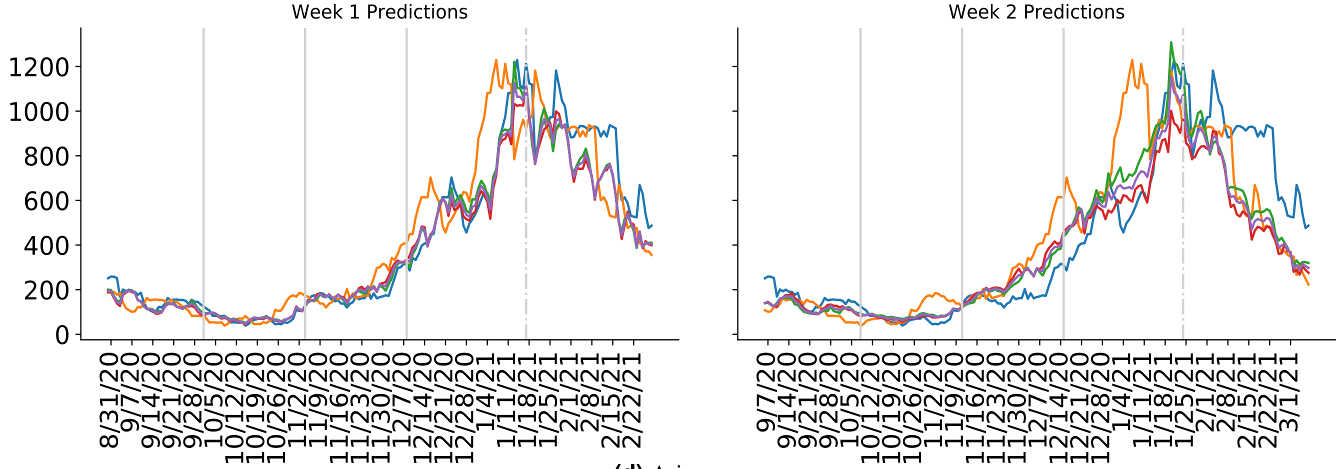

(d) Arizona

Figure S1. Weekly total number of deaths for Week 1 (left) predictions and Week 2 (right) predictions for Illinois, California, Texas, and Arizona. Vertical lines separate different prediction periods as in Table 2 in main article. The last dashed vertical line marks the prediction period of recent data using our last trained model. "Target" is the true reported number of deaths for the corresponding state.
